# Recent Advances in the Integrative Taxonomy of Plants

**DOI:** 10.3390/plants12244097

**Published:** 2023-12-07

**Authors:** Yevhen Maltsev, Andrey Erst

**Affiliations:** 1 K.A. Timiryazev Institute of Plant Physiology Russian Academy of Sciences, IPP RAS, Moscow 127276, Russia; ye.maltsev@gmail.com; 2 Central Siberian Botanical Garden, Siberian Branch, Russian Academy of Sciences, Novosibirsk 630090, Russia

Biodiversity conservation and management call for rapid and accurate global assessments at the species level [1]. At the same time, the rapid development of evolutionary biology based on a spectrum of approaches to test species relationships and species limits has revolutionised and is still revolutionising the science of plant systematics, including taxonomy [2]. Even if debate remains about the hierarchy of the types of characters and criteria to use for species delimitation, most taxonomists agree that objectively evaluating several lines of evidence within a formalised framework is the most efficient and theoretically grounded approach to defining robust species hypotheses [3,4]. A taxonomic workflow integrating multiple lines of evidence is proposed to facilitate the subsequent formal species description for plants, accommodating most species concepts, delimitation criteria, and data analysis methods. Species complexes are groups in which species limits and hence species numbers are unclear [5,6]. The integration of various data can be useful in solving hybridisation issues, describing new taxa, studying cryptic species, and identifying synonyms among the entire biological diversity of previously described species [7,8,9,10]. This Special Issue of *Plants* provides an excellent opportunity for the evaluation of new findings and experiences in the integrative taxonomy of plants (Introduction to the Special Issue “Integrative Taxonomy of Plants” in *Plants*).

At the present stage, most of the studies on microalgae and cyanobacteria are based on their precise species identification [11,12,13]. Such accuracy can only be achieved with an integrative approach, using classical (morphological and cultural) and modern molecular phylogenetic, biochemical, ecological, and other methods [8,12,14,15,16,17]. Moreover, the integrative approach can be interpreted in several directions. First, it can be used to assess the overall species richness and diversity of algae in different ecosystems and to interpret ecology and biogeography. This approach employs the analysis of morphological, ultrastructural, biochemical, physiological, ecological, and molecular data [18,19]. Second, the term ‘integrative taxonomy’ implies the description of new taxa with the simultaneous study of phenotypic, chemotaxonomic, and genotypic features [20,21]. At the same time, the authors of this Special Issue used all the potential of integrative taxonomy.

The Special Issue “Integrative Taxonomy of Plants” presents studies that highlight modern approaches to the integrative taxonomy of plants, algae, and fungi. This Special Issue contains seventeen papers, the majority comprising articles (fifteen papers, including one feature paper), followed by one review and one communication. A substantial amount of the articles reports new diatom species obtained using the integrative taxonomic approach. Based on molecular and morphological investigations, *Sellaphora terrestris* Glushchenko, Kezlya, Maltsev et Kulikovskiy were described from soil samples from Cát Tiên National Park (Vietnam) [15]. In this work, the authors discussed the systematic position of the diatom genus *Microcostatus* J.R. Johansen et J.C. Sray, since the new species was morphologically similar to representatives previously identified as *Microcostatus* species [22,23]. The aim of the next publication was the morphological and molecular study of *Cymbella himalaspera* Jüttner et Van de Vijver and new species *Cymbella baicalaspera* Glushchenko, Kulikovskiy et Kociolek from the Transbaikal Area (Siberia) with remarks about the phylogenetic position of the giant representatives of genus *Cymbella* C. Agardh based on molecular data [16]. The next work provided light and scanning electron microscope observations of *Achnanthes joursacensis* Héribaud populations from Mongolia [24]. This species is known from fossils and is now distributed in the Holarctic [25]. *Planoplatessa* Kulikovskiy, Glushchenko et Kociolek, a new monoraphid diatom genus, was described based on the current analysis of *A*. *joursacensis*. The authors indicated that the *Planoplatessa* species does not share main morphological features with *Achnanthes* Bory, *Planothidium* Round et Bukhtiyarova, and *Platessa* Lange-Bertalot representatives [26,27]. In addition, sixteen *Gomphonema* Ehrenberg species, including five new to science, were described from a shallow bay on the eastern shore of Lake Baikal [28]. This work presented information about the ecology and distribution of these *Gomphonema* species.

Another interesting work included the description of the chrysophycean new species *Chrysosphaerella septentrionalis* Kapustin from a peat bog near the Paz River (Pasvik Nature Reserve) using a scanning electron microscope [29]. *C. septentrionalis* is the first pseudocryptic species within the *C*. *longispina* Lauterborn complex [30], from which it differs by the presence of subcircular scales. In addition, the authors provided a formal description of a new family, Chrysosphaerellaceae. A new chlorophycean species of the genus *Mychonastes* P.D. Simpson et S.D. Van Valkenburg was described from the Moskva River [31]. The analysis was based on morphological characters, the SSU rDNA phylogenetic analysis, and ITS2 secondary structure. The authors concluded that *Mychonastes hindakii* Martynenko, Gusev, Kapustin et Kulikovskiy belongs to a cryptic species with differences only at the genetic level. Similar conclusions were obtained for green algae from the genus *Scenedesmus* Meyen [32]. The use of the genomic approach was one of the topics of the Special Issue. Genomic analysis, along with the 16S rDNA phylogeny, was used for the re-evaluation of the genetic diversity of 80 morphologically similar strains of cyanobacterial genera *Synechococcus* Nägeli, and *Parasynechococcus* F. Coutinho, Tschoeke, F. Thompson et C. Thompson [33]. One publication contains a renewed checklist of the algal marine flora of Vietnam, including 878 species of Cyanobacteria, Rhodophyta, Ochrophyta, and Chlorophyta [34]. The article reviewed the molecular-assisted alpha taxonomy of marine algae and examined algal biodiversity spatial patterns across Vietnam and the South China Sea region as a whole.

A promising biotechnological direction is the study of algal-derived organic matter from biopolymers (polysaccharides and proteins), refractory compounds (humic-like substances), low-molecular-weight acid and neutral compounds [35,36]. The investigation of algal fluorescent fraction in dissolved organic matter in the Special Issue was presented by Lobus et al. [37]. The authors analysed five strains of microalgae from the Ob and Yenisei Gulfs using molecular morphological investigations, and excitation–emission matrix fluorescence spectroscopy. The obtained results confirmed the feasibility of the production of a humic-like fluorescent fraction of dissolved organic matter in the Arctic shelf regions.

In addition to algae, the Special Issue examined and discussed such taxa as *Primula* L. (Primulaceae Vent.), *Riccia* L. (Ricciaceae Rchb.), *Nitraria* L. (Nitrariaceae), *Phyteuma* L. (Campanulaceae Juss.), *Rosa* L. (Rosaceae Juss.), *Tillandsia* L. (Bromeliaceae Juss.), and *Axyris* L. (Amaranthaceae Juss.). Conventional morphological methods were applied, along with chemophenetics, chemotaxonomical, ecological metabolomical, phylogenetics, bioimaging, sequencing, FAIR data, flow cytometry, palynology, biogeography, ddRAD, ecological niche modelling, and geometric morphometry.

Multilocus data analysis performed using a complex of methods revealed the diversity of cryptic species in the *Tillandsia ionantha* Planchon (Bromeliaceae) [38]. Phylogenetic analysis showed that *T. ionantha* is polyphyletic and composed of eight evolutionary lineages. Haplotype distribution and genetic differentiation analysis detected a strong population structure and high values of genetic differentiation among populations. A positive correlation between genetic differences and geographic distance indicates that the populations are evolving under the model of isolation by distance. The coalescent multispecies analysis supports the recognition of eight lineages as different species [39]. Only three out of the eight species have morphological characters good enough to recognise them as different species, while five of them are cryptic species. *Tillandsia scaposa* (L.B. Sm.) Ehlers and *T. vanhyningii* (B.T. Foster) Beutelspacher et García-Martínez are corroborated as independent lineages, and *T. ionantha* var. *stricta* Koide changed the status to the species level [38].

A new species, *Nitraria iliensis* Banaev et Tomoshevich, was described from the Ili basin, Almaty region, Kazakhstan [9]. It belongs to section *Nitraria* ser. Sibiricae is morphologically similar to *N. sibirica* Pall [40]. An integrative taxonomic approach based on molecular, biochemical, and morphological analyses, along with palynological data, was used to delimit this new species. 

The numeric analysis of colour was carried out, providing colorimetric variables together with the detailed description of the metabolic profiles of populations with different flower colours of species of the genus *Phyteuma* [41]. The data obtained allowed authors to show a unique chemical fingerprint that identifies species and subspecies with clear markers [42]. As a result, the taxonomic statuses of sympatric *P. spicatum* L., *P. ovatum* Honck., and *P. persicifolium* Hoppe were clarified.

*Rosa penduline* L., *R. spinosissima* L., and their hybrid *Rosa pendulina* × *spinosissima* were studied using a complex of methods, including morphometric analysis, flow cytometry, and liquid chromatography and mass spectrometry (HPLC-MS) 13 [43]. The complex analysis showed that the content of phenolic compounds in petals decreased after crossing, which confirms the native hybridisation process [44]. The content of flavanols and flavonols was lowest in the hybrid petals, whereas the content of anthocyanins was highest in the hybrid petals.

European *Leucanthemum* Mill. species are a taxonomically complex group of plants [45]. Therefore, to distinguish morphotypes and study the evolution of this group, an integrative approach was used which included RADseq data (consensus clustering), morphometrics of reconstructed leaf silhouettes from digitised herbarium specimens, and the quantification of species distribution overlaps [46]. The study showed that 17 of the 20 *Leucanthemum* morpho-species are supported by genetic evidence. The taxonomic rank of the remaining three morpho-species was resolved by combining genealogic, ecologic, geographic, and morphologic data.

Liquid chromatography high-resolution mass-spectrometry (UPLC/ESI-QTOF-MS) with data-dependent acquisition (DDA-MS) was integrated with DNA-marker-based sequencing of the *trn*L-*trn*F region, and high-resolution bioimaging was used to study the taxonomy of *Riccia glauca* L., *R. sorocarpa* Bisch. and *R. warnstorfii* Limpr. ex Warnst. with *Lunularia cruciata* (L.) Dumort. ex Lindb. The applied integrative approach revealed many chemophenetic markers of different complexity that can provide more mechanistic insights into the phylogenetic delimitation of species within a clade compared to genetic-based methods coupled with conventional morphology-based information [47].

Biogeography and systematics of the genus *Axyris* were based on a complex of methods [48]. A detailed study of the Tian Shan and Pamir Mountains, and the Himalayas and Tibet in herbaceous backgrounds, and the study of anatomy with the phylogenetic analysis, revealed that the loosely related *A. sphaerosperma* Fisch. et C.A. Mey. and *A. caucasica* (Sommier et Levier) Lipsky had the thickest seed coat among all Chenopodiaceae, and these traits probably evolved as adaptations to extremely low winter temperatures. This reproductive feature may explain the disjunct range of *A. sphaerosperma*, which is restricted to harsh climatic conditions [49].

Despite the widespread use of methods such as chemophenetics, ecological metabolomics, phylogenetics, flow cytometry, palynology, biogeography, and ecological niche modelling, which provide extensive information for analysis, conventional morphological methods are still relevant in hotspots with a high level of taxonomic diversity, including new species [50]. Recently, a new species, *Primula luquanensis* Z.K.Wu et Wei Zhou (Yunnan Province, China), was described and illustrated using classical taxonomy. The new species also has a substantially reduced corolla tube, presenting a unique floral form in the genus where heterostyly typically prevails [51]. However, in the case of complex taxonomy, with a large number of similar morphotypes, cryptic species, and hybridisation, the use of an integrative approach is justified and can help to identify and delimit taxa and their classification.

## Data Availability

The data presented in this study are available on request from the corresponding author.
